# Acute Diarrhea in Children after 2004 Tsunami, Andaman Islands

**DOI:** 10.3201/eid1505.081096

**Published:** 2009-05

**Authors:** Subarna Roy, Debdutta Bhattacharya, S.R. Ghoshal, K. Thanasekaran, A.P. Bharadwaj, M. Singhania, A.P. Sugunan

**Affiliations:** Indian Council of Medical Research Regional Medical Research Centre, Port Blair, Andaman and Nicobar Islands, India (S. Roy, D. Bhattachaya, S.R. Ghoshai, K. Thanasekaran, A.P. Sugunan); G.B. Pant Hospital, Port Blair (A.P. Bharadwaj, M. Singhania )

**Keywords:** Enteric infections, diarrhea, tsunami, disaster, Andaman Islands, letter

**To the Editor**: The Andaman Islands, population ≈350,000, are a territory of India located in the Bay of Bengal, northwest of Indonesia. On December 26, 2004, these islands were struck by an earthquake measuring 9.1 on the Richter scale (*1*) and by the ensuing Great Asian Tsunami (*2*). The fault slip, which caused permanent land subsidence of several meters (*3*) and ingression of sea water, resulted in the displacement of most survivors, many of whom were forced to live in temporary camps on higher ground for periods of more than a year. About 80% of the water supply lines were broken (*4*) and so were most sewage lines, making the situation ideal for transmission of water-borne diseases.

Because an outbreak of cholera had occurred in the Andaman and Nicobar Islands in 2002 (*5*), we were apprehensive about outbreaks of infectious diseases after the tsunami, particularly among children, who are less immune to most infections; therefore, we increased our efforts to identify and contain these possible outbreaks as quickly as possible. However, except for a cluster of cases of rotaviral diarrhea (*6*), no major infectious disease outbreak occurred among residents of the Andaman Islands in the year that followed the tsunami.

Although the incidence of severe cases of diarrhea among children admitted to G.B. Pant Hospital in Port Blair, the only referral hospital in the Andaman Islands, varied greatly from month to month during 2001–2007, the incidence began decreasing after 2005, as indicated by the 12-month moving average (Figure). The mean number of cases per year fell from 361.4 during 2001–2005 to only 255.0 during 2006 and 2007 (p = 0.00025).

**Figure:**
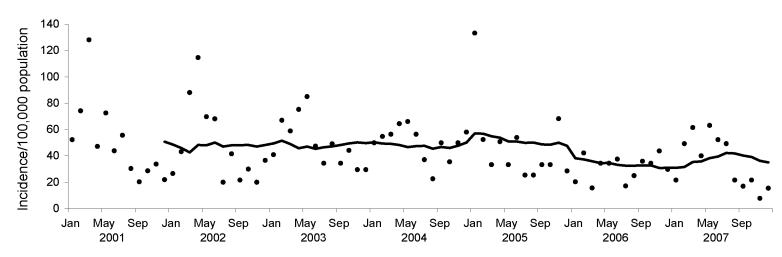
Estimated monthly incidence (black dots) of acute diarrhea among children <15 years of age in the Andaman Islands and 12-month moving average of the monthly incidence (black line), 2001–2007. Data based on cases of disease among children admitted to G.B. Pant Hospital, Port Blair, Andaman and Nicobar Islands, India.

The estimated annual incidence of acute diarrhea per 100,000 children in the Andaman and Nicobar Islands was 609 in 2001, 580 in 2002, 595 in 2003, 601 in 2004, 571 in 2005, 370 in 2006, and 420 in 2007. For these incidence estimates, the population at risk during the years 2002–2007 was calculated by extrapolating from the 2001 census population on the basis of an annual population growth rate of 1.53% (the average for 1991–2001) and assuming that children <15 years old constituted 36.2% of the total population each year (as they did in 2001). The reduction in the number of acute cases of childhood diarrhea began several months after the tsunami, when the water and sewage systems of the islands had been repaired and renovated in many areas.

According to official reports, the cost of the restoration and renovation of the water and sewage systems after the tsunami was 389.9 million rupees, >2× the projected cost of work on the water and sanitation systems (172.9 million rupees) prior to the tsunami (*4*). In the aftermath of the tsunami, 52 km of new pipelines were laid and 12.5 km of old pipelines were replaced. Water supplies were augmented in 49 areas (*4*). The revamped water and sewage systems eliminated many sources of fecal contamination.

Moreover, by the middle of 2005, post-disaster assistance had been provided by voluntary organizations, missionaries, nongovernmental organizations, and government agencies from mainland India and abroad. This assistance resulted in further improvements in the area’s public sanitation infrastructure and hygiene, particularly in the temporary shelters that displaced residents were living in; it also raised awareness among island residents about the threat of water-borne diseases. All of these factors were likely contributors to the decline in the number of cases of acute diarrhea in children after the tsunami. Although out-migration of island residents or a reduction in case detection after the tsunami also could have contributed to the observed decline in cases of diarrhea, no large-scale migration was reported during the period, and disease surveillance systems were in fact strengthened after the tsunami and further strengthened with the introduction of the Integrated Disease Surveillance Program.

In summary, we found that the incidence of acute diarrhea among children of the Andaman Islands decreased within months after the 2004 tsunami. This result highlights the importance of public health and sanitation measures after a natural disaster.
